# DNA Binding and Condensation Properties of the Herpes Simplex Virus Type 1 Triplex Protein VP19C

**DOI:** 10.1371/journal.pone.0104640

**Published:** 2014-08-14

**Authors:** Alakesh Bera, Edward M. Perkins, Jian Zhu, Heng Zhu, Prashant Desai

**Affiliations:** 1 Viral Oncology Program, The Sidney Kimmel Comprehensive Cancer Center at Johns Hopkins, Johns Hopkins University, Baltimore, Maryland, United States of America; 2 Department of Biology and Integrated Imaging Center, Johns Hopkins University, Baltimore, Maryland, United States of America; 3 HiT Center and Department of Pharmacology and Molecular Sciences, Johns Hopkins University, Baltimore, Maryland, United States of America; University of Minnesota, United States of America

## Abstract

Herpesvirus capsids are regular icosahedrons with a diameter of a 125 nm and are made up of 162 capsomeres arranged on a T = 16 lattice. The capsomeres (VP5) interact with the triplex structure, which is a unique structural feature of herpesvirus capsid shells. The triplex is a heterotrimeric complex; one molecule of VP19C and two of VP23 form a three-pronged structure that acts to stabilize the capsid shell through interactions with adjacent capsomeres. VP19C interacts with VP23 and with the major capsid protein VP5 and is required for the nuclear localization of VP23. Mutation of VP19C results in the abrogation of capsid shell synthesis. Analysis of the sequence of VP19C showed the N-terminus of VP19C is very basic and glycine rich. It was hypothesized that this domain could potentially bind to DNA. In this study an electrophoretic mobility shift assay (EMSA) and a DNA condensation assay were performed to demonstrate that VP19C can bind DNA. Purified VP19C was able to bind to both a DNA fragment of HSV-1 origin as well as a bacterial plasmid sequence indicating that this activity is non-specific. Ultra-structural imaging of the nucleo-protein complexes revealed that VP19C condensed the DNA and forms toroidal DNA structures. Both the DNA binding and condensing properties of VP19C were mapped to the N-terminal 72 amino acids of the protein. Mutational studies revealed that the positively charged arginine residues in this N-terminal domain are required for this binding. This DNA binding activity, which resides in a non-conserved region of the protein could be required for stabilization of HSV-1 DNA association in the capsid shell.

## Introduction

The herpesvirus particle consists of four distinct structural layers. The outermost layer is an envelope in which are embedded the virus glycoproteins; the envelop encloses the tegument layer which is attached to the capsid, the viral protein coat; and the capsid encases and protects the virus genome [1], [2]. The capsid is an icosahedron made of four shell proteins that lie on a T = 16 icosahedral lattice [3]. These proteins are the major capsid protein, VP5, the triplex proteins, VP19C and VP23 and the small capsid protein, VP26 [4], [5]. The shell also contains four structural elements the hexon, the penton, the triplex and the portal. The hexons and pentons are made up of VP5 (149 kDa). The triplex, which is a characteristic structural feature of all herpesvirus capsids, is a hetero-trimer of VP19C (50 kDa) and two molecules of VP23 (34 kDa). The triplex acts to stabilize and facilitate capsid shell synthesis by interaction with adjacent hexons or pentons. VP5, VP19C and VP23 are all essential for capsid assembly in HSV-1 infected cells [6]–[8].

**Figure 1:**
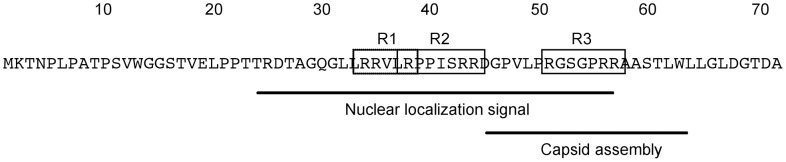
Amino acid sequence of the N-terminal domain (1–72) of HSV-1 VP19C. The R1-R3 boxes represent the three arginine-rich clusters. R1 box includes R34, R35 and R38; R2 box includes R38, R43 and R44 and R3 box encompasses R51, R56 and R57. Adamson *et al*. [7] identified a domain between amino acids 45 and 63; that is essential for capsid assembly and virus growth. The nuclear localization signal is present in a 33 amino acid sequence within the N-terminal 56 residue region [7], [16].

The interaction between VP19C and VP23 has been inferred by cryo-EM studies [9]–[11] and confirmed using data from yeast two-hybrid [12], [13], co-sedimentation [14] and co-localization experiments [7], [15]. One of the functions of this interaction is the nuclear translocation of VP23 by VP19C and a study by Adamson *et al*. [7] has identified a non-consensus nuclear localization sequence (NLS) at the N-terminus of this protein. Additional functional domains of both VP19C and VP23 have been identified using random transposition [7], [8] and deletion mutagenesis [7], [14]. The results from these studies indicate the presence of multiple interaction domains in both of these molecules that are required for triplex formation and consequently capsid assembly. Although the N-terminus of VP19C is not conserved in the other herpesvirus sub-families, it is functionally important for alphaherpesvirus capsid assembly and maturation. Deletion mutations of VP19C revealed the presence of a NLS [7], which maps to a 33-amino-acid region in the N-terminal 56 amino acid region of VP19C (Fig. 1). Adamson *et al*. [7] also demonstrated that the first 45 amino acids of VP19C are not essential for assembly of functional capsids and infectious particles but deletion of the N-terminal 63 amino acids resulted in formation of aberrant capsids and prevented virus growth (Fig. 1).

Furthermore, recent studies have confirmed the presence of non-classical NLS in VP19C between aa 50 and 61, and nuclear import of VP19C was mediated by RanGTP and an importin beta1-, but not importin alpha5-, dependent pathway [16]. Additional studies have also shown the presence of a nuclear export signal (NES) between aa 342 to 351 which enable VP19C to shuttle between the nucleus and the cytoplasm [17]. These signals are important for virus replication and production [16], [18]. The presence of both the NLS and NES in VP19C is functionally similar to the Rev protein of HIV-1 [19]. These studies indicate that VP19C is a multifunctional protein and may have additional roles during virus maturation besides triplex formation.

Analysis of the primary sequence (Table 1) revealed that VP19C is highly basic (pI 9.06), and glycine-rich. This analysis also showed that the majority of the positive charge, which is contributed predominantly by arginine residues in VP19C, is localized in the first 72 amino acids (pI 11.7) of VP19C. This domain is also structurally more flexible due to a high glycine content (12.5%). Therefore we hypothesized that the N-terminal 72 amino acid sequence of VP19C might bind to nucleic acids. The C-terminal domain of the protein (73–465 amino acids) is comparatively less positively charged and structurally less flexible (8.4% glycine) and has five short helices (5 to 8 Å) [11]. Previous studies by Braun *et al*. [20] showed VP19C could bind to DNA. In addition it was also shown that fusion of a VP19C N-terminal domain to β-galactosidase not only interfered with virus replication but also localized β-galactosidase to virus DNA replication compartments [21]. These data prompted us to investigate the DNA binding property of VP19C. The results indicate that VP19C can bind non-specifically to dsDNA but not to single stranded DNA (ssDNA). In addition, analyses of nucleo-protein complexes by electron microscopy revealed that VP19C could change the conformation of linear DNA into a condensed toroidal structure. These functional activities of VP19C reside in the N-terminal 72 amino acid region of this molecule. This novel property of VP19C may have biological significance for HSV-1 capsid assembly and DNA encapsidation.

**Table 1:** Amino acid sequence analysis of HSV-1 VP19C.

protein	arg+lys (+ve)	asp+glu (-ve)	gly	pI
VP19C (1–465 aa)	52	43	9.0%	9.06
N-ter (1–72 aa)	10	5	12.5%	11.70
C-ter (73–465)	42	38	8.4%	8.50

## Materials and Methods

### Plasmids

The VP19C (UL38) ORF was PCR amplified using KOS genome DNA as a template. The forward and reverse primers specified yeast homology sequences for recombination (Table 2). Recombination in yeast was performed as described in Hudson *et al*. [22]. The yeast expression plasmid was designated pEGHUL38. In this plasmid the protein synthesized will contain an N-terminal glutathione S-transferase sequence (GST)-6XHIS tag for purification [23]. The sequence specifying the N-terminal 72 amino acids of VP19C (VP19C N-ter^72^) was PCR amplified using pBS19C [24] as a template and cloned as an EcoR1-Spe1 fragment into the same sites of a modified pGEX4T3-Spe1 (GE Healthcare) vector. This plasmid (pGEX4T3) contains the GST domain followed by a thrombin cleavage site sequence and the multiple cloning site. All PCR amplifications were done with *Pfu* or *Pfu* Ultra polymerases (Stratagene). Amino acid substitutions of the arginine residues to alanine in the N-terminus of VP19C (Fig. 1) were generated using QuikChange (Stratagene) methods using pKUL38Spe1 [25] as a template and the primers documented in Table 2. In each QuikChange cycle, the arginines in three arginine-rich boxes (R1, R2 and R3, see Fig. 1) were mutated to alanine. Different combinations of the R-rich box mutations were made by using one of the mutant plasmids as a template (for example R1A) and primers specifying a mutation in another R box (for example R3A) to give mutant R1R3A. The primers used for mutagenesis are shown in Table 2. All mutations were transferred into pGEX4T3-Spe1 using the same PCR amplification and cloning procedure performed for VP19C N-ter^72^. The wild-type and mutant plasmids were designated pVP19C N-ter^72^, and pVP19C N-ter^72^R1A, etc. All PCR amplified DNAs and mutant plasmids were sequenced for authentic amplification.

**Table 2:** Primers and oligonucleotide sequences used in this study.

Name	Primer Sequence	Application
UL38pEGH/F	a *caccatcacggtggtggt*atgaagaccaatccgctaccc	Yeast
UL38pEGH/R	a *agtcagtcacgatgaatt*tcacgcgcatgcccgccactc	Yeast
UL38pGEX/F	b *ggaattc*catgaagaccaatccgctaccc	E.coli
UL38pGEX/R	b *ggactagt*tcacgcgtctgtgccgtccaggcc	*E.coli*
R1A F	c gggcagggcctgcttgctgcagtcctggcccccccgatctcgccg	QuikChange
R2A F	c cttcggcgcgtcctggcccccccgatctctgcagccgacggccag	QuikChange
R3A F	c ggcccagtgctcccggcgggatccggacccgcggcggcggcaag	QuikChange
DNA (66mer)	Ccccccaccacacgcgatactgcagggcagacgctgtggttgcttggcctggacggcacagacgcg	EMSA
DNA (28mer)	Ggaattcatgaagaccaatccgctaccc	EMSA

a yeast homology sequences are italicized.

b restriction sites are underlined and italicized.

c alanine substitutions are underlined.

### Gel electrophoresis

Sodium dodecyl sulfate-polyacrylamide (SDS-PAGE) analysis was performed as described in Person and Desai [24]. In some experiments NuPage (Invitrogen) gels were used and the manufacturers protocol was followed for electrophoresis of purified proteins using MES buffer (Invitrogen).

### Purification of the VP19C protein from Yeast

**Figure 2:**
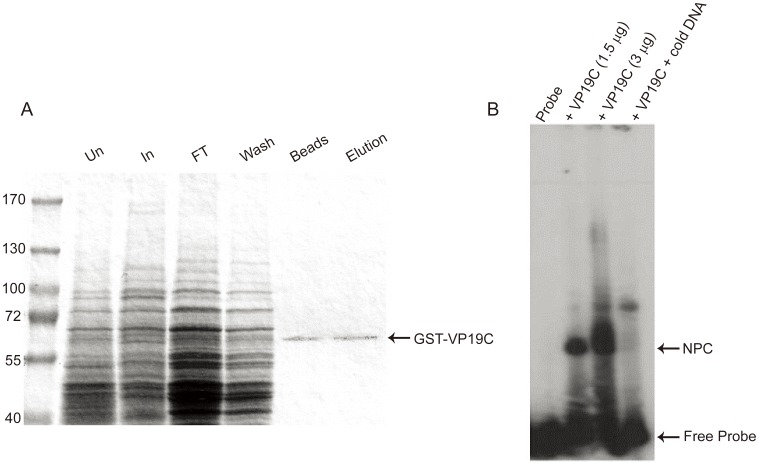
Purification of VP19C and the DNA-binding property of the purified protein. A. Expression and purification of VP19C protein from yeast cells. Yeast cells induced for expression of GST-VP19C and the proteins purified from these cells were analyzed by SDS/PAGE using 4-12% Nu-PAGE gels and detected by Coomassie Blue staining. Total proteins extracted from uninduced cultures (Un) and cells induced (In) with glucose are shown. The protein fractions, flow-through (FT) and wash (Wash) obtained during the purification are also shown. GST-VP19C bound with glutathione–Sepharose beads before elution (Beads) and the eluted purified protein (Elution) were detected. Molecular-mass markers; lane 2. B. VP19C specifies a DNA-binding property. In the EMSA a small dsDNA (66 bp) radiolabeled probe (1.5 ng) was incubated with 1.5 µg (lane 2) and 3 µg VP19C (lanes 3 and 4) or no protein (lane 1). In lane 4, a 50-fold excess of non-labeled DNA was added to the VP19C-DNA composition. The nucleo-protein complex (NPC) and the location of the free probe are indicated on the left of the panel.

Full length VP19C (tagged with GST) was purified from yeast using protocols described in Casamayor and Snyder [26] and Zhu *et al*. [23]. Briefly, a fresh colony was used to inoculate SC (-uracil plus glucose) media and incubated overnight at 30°C. The following day, the OD of the culture was measured and a one litre culture in SC (-uracil plus raffinose) was started with an OD_600_ = 0.0125. When the OD_600_ reached 0.6–0.8 (approximately 16 h later) the protein expression was induced using a final concentration of 2% galactose. After 6 h of growth, yeast cells were collected by centrifugation and washed one time in cold water, and cell pellets were kept frozen at −80°C. Fusion proteins were purified at 4°C as follows. The cell pellet was resuspended in 20 ml purification buffer (Dulbeccos's PBS, 50 mM EDTA and Complete protease inhibitor cocktail [Roche]) together with 1 ml of zirconia glass beads (0.5 mm diameter). Cells were disrupted by vortexing at maximum speed for 10 min at 4°C. The lysate was clarified by centrifugation for 5 min at 13,000 *g*, and the protein extract was kept on ice. The extract was mixed with 1 ml of Glutathione Sepharose-4B beads (GE-Healthcare) equilibrated in purification buffer and binding was carried out for 2 h at 4°C with rocking. Proteins bound to the Glutathione Sepharose-4B beads were washed 3 times with 20 ml purification buffer containing 0.5 M NaCl and once with cold DPBS. Protein was eluted from the beads using elution buffer (DPBS with 5% glycerol) containing 15 mM reduced glutathione. Purified proteins were dialyzed in storage buffer (10 mM Tris pH 7.4, 50 mM NaCl and 1% glycerol). The purity of the protein was examined by gel electrophoresis and Coomassie blue staining and determined to be >95% (Fig. 2A).

### Purification of the wild type and mutant of VP19C N-ter^72^ from *E.coli*

Sequences encoding the N-terminal 72 amino acids of VP19C (VP19C N-ter^72^) were cloned into pGEX4T3 for purification using GST affinity chromatography. The plasmids were transformed into *E.coli* BL21-CodonPlus (DE3)-RIPL cells (Stratagene). Overnight cultures were diluted 1∶100 into 500 ml of 2YT growth medium, and protein expression induced with 0.1 mM IPTG when the culture OD_600_ reached approximately 0.6 at 37°C. Induced cultures were incubated an additional 2 h at 37°C, harvested by centrifuging the cells at 5000 rpm for 15 min at 4°C. The cell pellet was then washed with ice cold 10 ml DPBS once, resuspended in STE buffer (10 mM Tris-HCl pH 8; 250 mM NaCl, 25 mM EDTA) containing Complete protease inhibitor cocktail. Cells were freeze-thawed three times and sonicated for 30 sec for two times. The total homogenate was clarified by centrifuging at 15,000 *g* at 4°C for 30 min. The lysate was then mixed with a 50% slurry (1.5 ml) of Glutathione Sepharose-4B beads and incubated at 4°C for two hours with constant end-to-end rotation. The beads were washed with 5 ml STE buffer two times, then washed with 5 ml buffer B (STE containing 0.5% Triton-X100) followed by 5 ml Buffer C (STE containing 1 M NaCl) and finally washed again with 5 ml DPBS containing 5% glycerol. The GST-tagged VP19C-N-ter^72^ was eluted off from the beads using elution buffer containing 15 mM reduced glutathione in DPBS with 5% glycerol. Proteins were finally dialyzed in storage buffer (10 mM Tris pH 7.4, 50 mM NaCl and 1% glycerol). Both wild type and mutant polypeptides were more than 90% pure as judged by Coomassie staining SDS-PAGE gels.

### Electrophoretic mobility shift assay (EMSA)

For the gel shift assay, DNA probes from HSV-1 and non-HSV-1 sequences were used. They were: BamH1 K (5.9 kb) fragment (derived from pSG1-EK1) [27]; pUC19 (linearized with EcoR1), a 66mer annealed oligonucleotide (dsDNA) and a 28mer oligonucleotide (ssDNA) (Table 2). The plasmid probes were first treated to antartic phosphatase to remove the 5′ phosphates. Purified plasmid DNA or oligonucleotide sequences were end-labeled using T4 polynucleotide kinase in the presence of [γ-^32^P] ATP. The radiolabeled probes were purified using Sephadex G50 (GE Healthcare) columns. Purified VP19C (GST-VP19C), VP19C N-ter^72^ wild type or mutants was mixed with ^32^P-labeled 5'-dsDNA or single stranded oligonucleotide probes for 10 min at 37°C in 10 µl of a nucleo-protein complex (NPC) formation buffer [20 mM Tris·HCl, pH 7.5, 30 mM NaCl, 0.2 mM MgCl_2_, 5 mM dithiothreitol, 0.01 mM ZnCl_2_]. Reactions were stopped by addition of 5 mM EDTA (final concentration) and the samples were electrophoresed on either 4% or 6% acrylamide gels in 0.5X TBE. Gels were dried and exposed to X-ray film for autoradigraphy.

### Negative stain preparation for transmission electron microscopy

Nucleo-protein complexes for ultrastructural analysis were made by mixing 250 ng of DNA with 150 µg of purified protein in 0.1 M ammonium acetate (pH 5), the final volume was 50 µl. After 15 min incubation 10 µl drops of the protein/DNA solutions were placed on freshly ionized carbon and formvar coated 400 mesh grids for between 1 and 3 minutes, blotted on filter paper, briefly floated on two drops of 2% uranyl acetate, followed by two drops of ddH_2_O, blotted on filter paper, and allowed to air dry. The samples were observed on Phillips EM410, EM420 and FEI Tecnai 12 transmission electron microscopes (FEI Co., Hilsboro, Oregon) operating at 100 kV. Images were recorded with SIS Megaview III digital cameras and Analysis 3.2 or iTEM 5.0 software (Olympus Soft Imaging Solutions Corp., Lakewood, CO). Protein/DNA complexes were measured with Analysis 3.2 and iTEM 5.0.

## Results

### DNA binding property of VP19C

As discussed above, sequence analysis of the N-terminus of VP19C revealed a region of the protein that could potentially bind to DNA (Fig. 1). This region also encodes a nuclear localization sequence [7] [16]. Previous studies by Braun *et al*. [20] demonstrated a DNA binding activity associated with VP19C. In that study capsid proteins were transferred to a nitrocellulose membrane and a radiolabeled probe corresponding to the junction fragment of HSV-1 bound to a polypeptide with the same mobility as VP19C. To confirm this DNA binding property of VP19C, an electrophoretic mobility shift assay (EMSA) was carried out. Previous attempts to purify full-length VP19C from *E.coli* were not successful. Thus, we used a yeast expression system to purify the VP19C polypeptide [23]. The yeast expression plasmid produces a GST-6XHIS-VP19C polypeptide for purification purposes. This approach was successful in purifying (>95% purity) full-length VP19C (GST-VP19C) protein for the gel-shift assay, Fig. 2A. In the first experiment a small dsDNA probe (66 bp) was used and incubated with VP19C and the nucleoprotein complexes analyzed by gel-shift methods (Fig. 2B). Radioactivity corresponding to a retarded DNA-protein complex was detected when VP19C was mixed with the probe (lane 2) indicating by this method VP19C does bind to DNA. Although there is a predominant band corresponding to the nucleo-protein complex (NPC), there is also additional smearing of radioactivity especially when more VP19C is used in the assay (lane 3). This could be due to the formation of different sizes of DNA-protein complexes arising from different stoichiometry of the protein-nucleic acid interaction between DNA and VP19C. When an excess (50 fold) of unlabeled DNA was added to the reaction mixture, the cold probe effectively competed for binding as judged by a reduction in the radioactivity corresponding to the DNA-protein complex in the gel (lane 4). This data indicated that VP19C does specifically bind to DNA.

**Figure 3:**
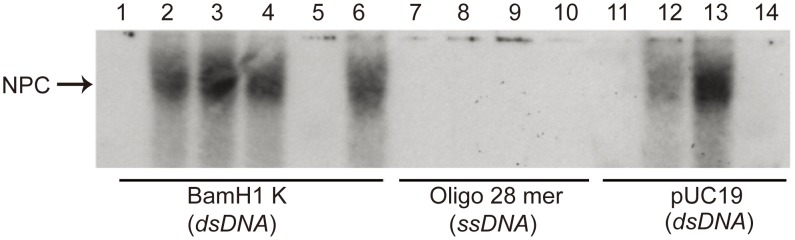
VP19C binds non-specifically to dsDNA probes. Three different nucleic acids were used in the EMSA; HSV-1 DNA (BamH1 K, 5.9 kb; lanes 1–6), plasmid pUC19 (2.7 kb; lanes 11–14) that was linearized by EcoR1 and an oligonucleotide (28mer; lanes 7–10) to test the specificity of the binding. All DNA molecules were 5′-end-labeled with [γ-^32^P] ATP. In lanes 1, 7 and 11, dsDNA or oligonucleotide probe alone was added to the assay (1.5 ng). In lanes 2-4 the DNA probe was incubated with increasing concentrations of VP19C (1.6 µg, 3.2 µg and 6.4 µg respectively) in NP-complex forming buffer. Similarly in lanes 8 and 12, 1.6 µg of VP19C was used and in lanes 9 and 13, 6.4 µg of VP19C was used. Lane 6 conditions were the same as lane 4 but the NP-complex forming buffer was free of ZnCl_2_. In lanes 5, 10 and 14 the DNA-probe was incubated with 6.4 µg of purified GST.

The dsDNA used in Fig. 2B was an annealed product of two complimentary 66mer oligonucleotides and the sequence was a VP19C N-terminal sequence. In order to test whether VP19C specifically binds HSV-1 DNA sequences or non-specifically binds DNA molecules, additional gel shift assays were performed. Two dsDNA molecules, one the L-S junction fragment of HSV-1 (BamHI K fragment) similar to that used by Braun *et al*. [20] and a bacterial plasmid (linearized pUC19) DNA, were used to test the specificity of the binding. In addition, a small oligonucleotide (28mer) was included to test whether VP19C could also bind a single-stranded DNA molecule. Nucleoprotein complexes were observed when VP19C was mixed with both the BamH1 K fragment as well as with pUC19 (Fig. 3, lanes 2–4 and 12–13) indicating VP19C does not require sequence specificity for DNA binding. It appears that increasing amounts of VP19C in the reaction with pUC19 (lanes 12–13) results in an increase in the radioactivity detected in the gel. This was not evident in the reaction with the BamH1 K fragment (lanes 2–4). DNA-protein complexes were not detected in the experiment, when a single-stranded molecule was used (lanes 8–9) indicating VP19C binds only double-stranded DNA molecules. NPCs were not observed when GST by itself was used in the assay with either of the three DNA molecules (lanes 5, 10 and 14), therefore, even though the VP19C protein used in this assay is GST tagged, this domain is not responsible for the activity observed in the gel shift assay. Because DNA-protein interactions are sensitive to the presence of heavy metal ions such as Mg^2+^ and Zn^2+^, the assay was done in the absence of Zn^2+^. As shown in lane 6, there is no effect of Zn^2+^ ion concentration on formation of the NPC (Fig 3).

**Figure 4:**
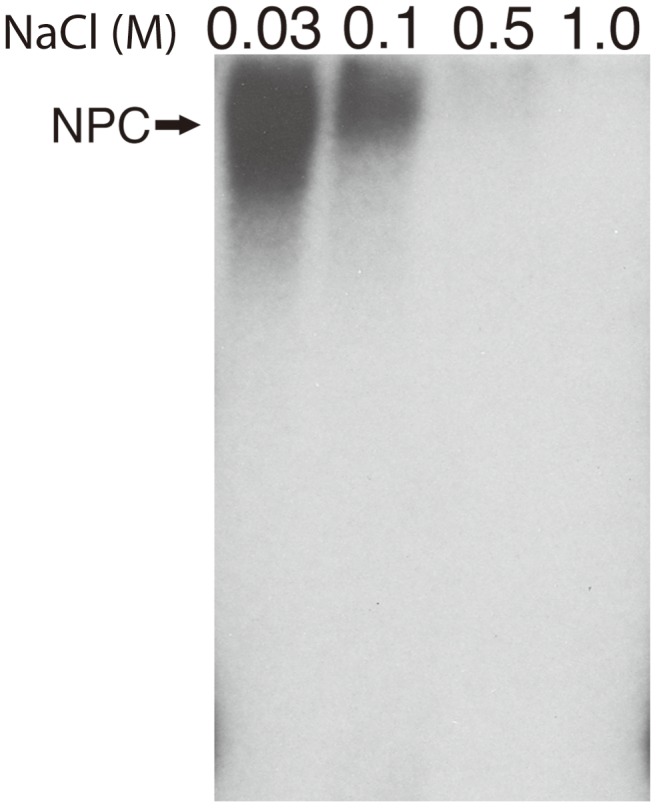
Effect of salt concentration on nucleo-protein complex formation. Purified VP19C (3 µg) was mixed with the BamH1 K radiolabeled probe (1.5 ng). The formation of a NPC was detected in the first lane when NP-complex forming buffer containing 30 mM NaCl was used for the assay. In similar experiments the NaCl concentration in the NP-complex forming buffer was increased to 0.1, 0.5 and 1.0 M. NPC formation was abolished completely in the presence of 1.0 M NaCl. An arrow on the left side of the panel indicates the VP19C-DNA NPC.

The effect of salt concentration specifically Na^+^ on the formation of the DNA-VP19C complex was also investigated (Fig 4). The result of the gel shift assay showed that increasing concentrations of NaCl reduced the amount of NPC formation as judged by the reduction in radioactivity detected in the gel (compare the first two lanes) and at concentrations greater than 100 mM (lanes 0.5 and 1.0) there is complete abolishment nucleoprotein complex formation between VP19C and the BamH1 K fragment. This result indicates that the interaction between protein and nucleic acid is due to electrostatic charge interactions between a positively charged region of VP19C and negatively charged DNA molecule.

### DNA binding by VP19C N-ter^72^

**Figure 5:**
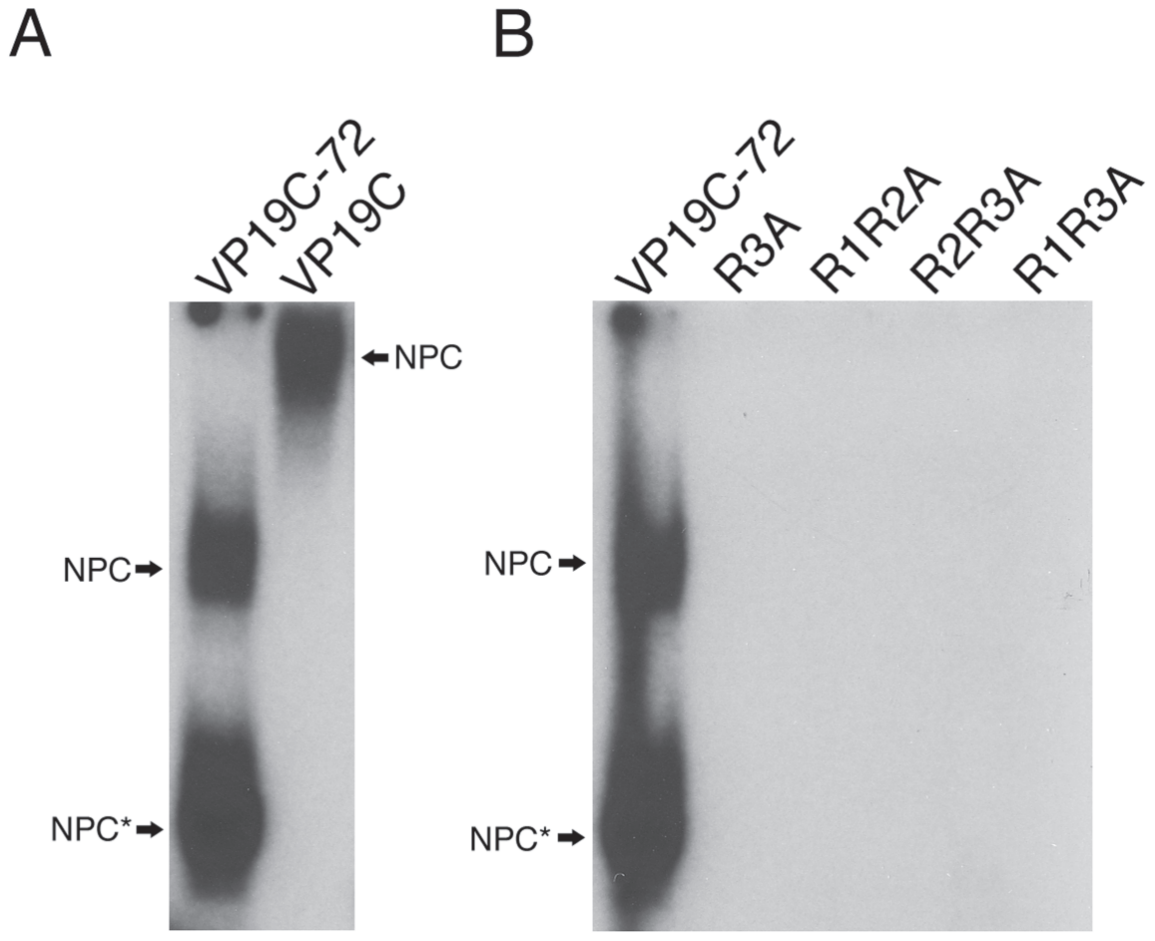
DNA binding activity of VP19C resides in the N-terminal 72 amino acids. A. Purified VP19C N-ter^72^ (6.4 µg) or full-length VP19C (3 µg) was mixed with the BamH1 K radiolabled probe (1.5 ng) and complex formation analyzed by EMSA. Two distinct NPCs were detected when VP19C N-ter^72^ was used. To compare NPC formation with the full-length VP19C, we performed EMSA with full-length protein (lane 2). B. Similar reactions were also performed with wild-type VP19C N-ter^72^ and mutant VP19C N-ter^72^ polypeptides specifying alanine-substitution mutations in the R-rich boxes (see Fig. 1). The arrows on the sides of panels A and B indicate the positions of the NPC in the gel.

The N-terminal domain of VP19C not only specifies a nuclear localization signal, but is also arginine-rich. There are 9 arginine residues present within the first 72 amino acids. We have determined that two residues, R55 and R56 are sufficient to act as an NLS (data not shown). This N-terminal domain, which we refer to as VP19C N-ter^72^ could therefore be the potential candidate for the DNA binding activity of VP19C. In order to examine the DNA binding properties of VP19C N-ter^72^, sequences encoding this region were cloned into an *E.coli* expression vector and the polypeptide was purified from cultures induced with IPTG. Although a soluble form of this N-terminal polypeptide was purified, when the purified protein was analyzed by SDS-PAGE followed by Coomassie staining, there was in addition to the full-length GST-VP19C N-ter^72^ a proteolytic degraded polypeptide (data not shown). Nevertheless, there was a significant population of VP19C N-ter^72^ that was not degraded and thus this protein mixture was used in the gel shift assay (Fig. 5A). A NPC was detected in the gel when both the full length VP19C and VP19C N-ter^72^ (VP19C-72) were incubated with the BamH1 K probe. The NPC detected when VP19CN-ter^72^ was used displayed a smaller retardation in the gel presumably because of the smaller size of the protein used (79 kD versus 36 kD which includes GST) and also two DNA-protein complexes were detected because of the two polypeptides (NPC: VP19C-72 and NPC*: proteolytic degraded) present in the VP19C N-ter^72^ protein mixture (Fig. 5A, first lane). These data demonstrate that the DNA binding domain of the VP19C resides in the N-terminal 72 amino acids.

In order to characterize the DNA-binding property of VP19C N-ter^72^ the arginine residues in the N-terminal fragment of VP19C were changed to alanine. As shown in Fig. 1 we divided the arginines into three arginine-rich boxes, R1 (R34, R35, R38), R2 (R38, R43, R44) and R3 (R51, R56, R57). The arginines in each box were changed to alanine and subsequently different combinations of the mutations were made, for example, R1R2A. These proteins were purified using the same procedures as before. The wild-type VP19C N-ter^72^ and the mutant polypeptides were tested for their ability to bind to the BamH1K junction fragment (Fig. 5B). Two DNA-protein complexes were detected in the gel for wild-type VP19C N-ter^72^ (Fig. 5B, first lane) as previously seen, however, none of the mutant polypeptides were able to bind DNA as judged by the absence of a retarded DNA probe (Fig. 5B) indicating that the arginines in the N-terminal 72 amino acid polypeptide are important. These data also show mutation of any R-rich box abolished NPC formation.

### DNA condensation by VP19C

**Figure 6:**
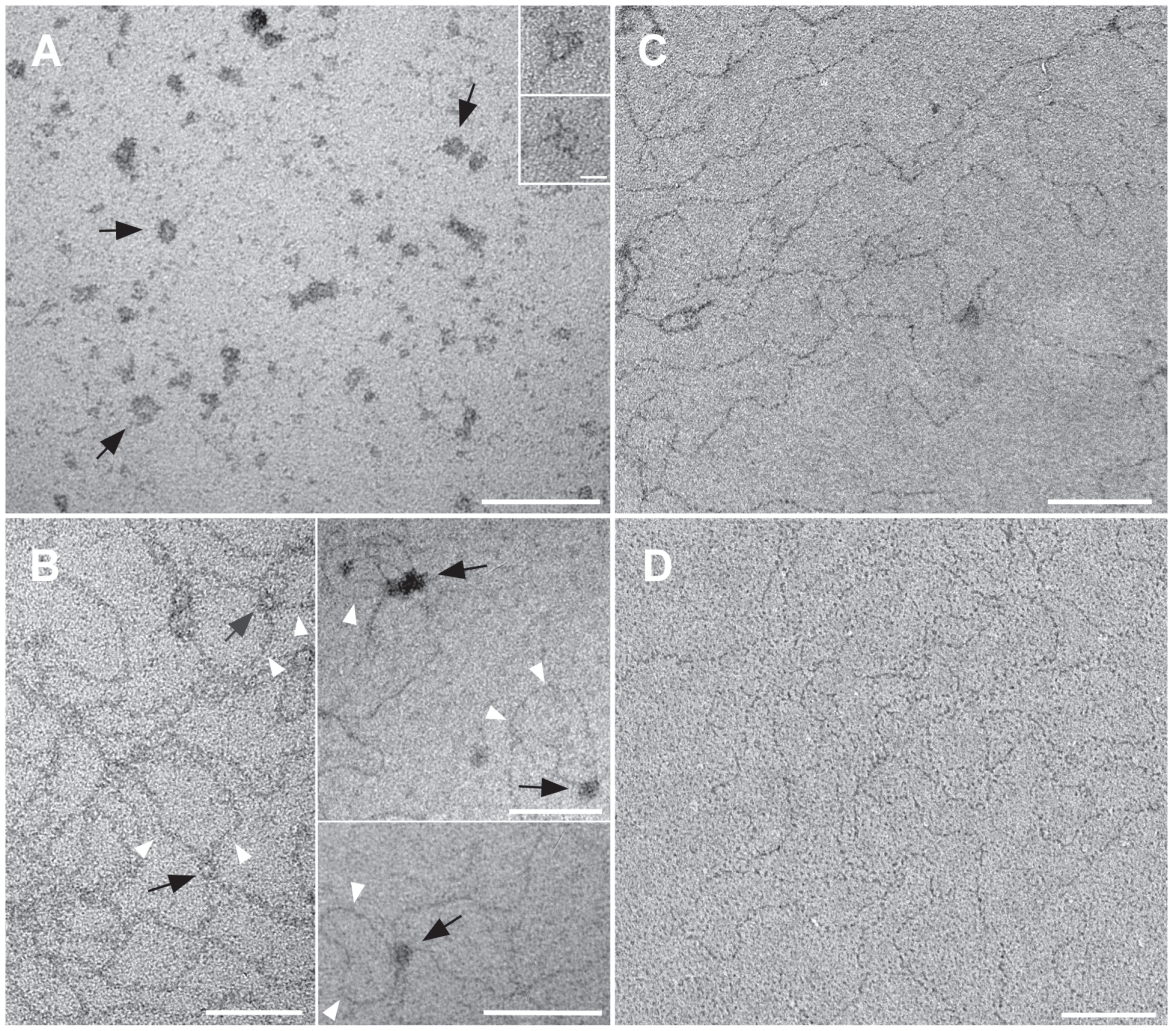
DNA condensation by VP19C. The morphology of the VP19C-DNA complexes was analyzed by electron microscopy. Purified VP19C (150 µg) was mixed with the BamH1 K DNA (250 ng) substrate and the NPC examined after 15 min of incubation. In panel A, full-length VP19C was mixed with DNA. Toroids of different sizes were detected and are indicated by black arrows. In panel B, VP19C N-ter^72^ was incubated with DNA. DNA loop structures (white arrowheads) along with aggregated condensed DNA (marked by black arrows) were induced by VP19C N-ter^72^. Shown in panel C and D is the morphology of DNA in the presence of GST protein or no added protein, respectively. Scale bars are 100 nm and 10 nm for panel A inset.

**Figure 7:**
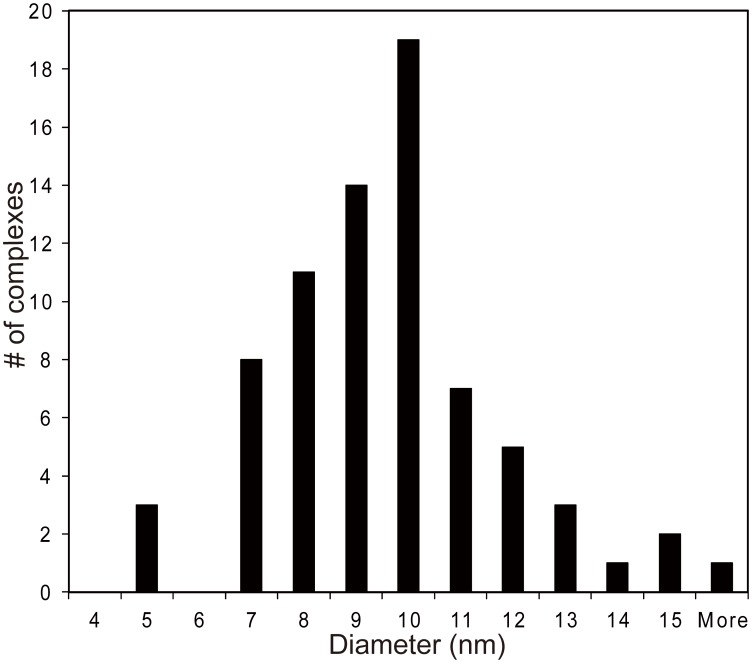
Distribution of the outer diameter of DNA toroids. Digital electron micrographs of toroids induced by full-length VP19C were examined and a histogram of the distribution of outer diameters of 74 measured complexes were plotted. The mean outer diameter of the toroids was 9.1 nm, with a standard deviation of 2.2 nm.

Some proteins that bind DNA can also condense DNA into a toroidal structure. This has been shown for an HIV structural protein [28] and it is also a characteristic property of spermines and magnesium metals [29]. An ultrastructural study was carried out on VP19C-DNA complexes to determine their structural configuration. Both the full length VP19C and VP19C N-ter^72^ were incubated with DNA (BamH1K fragment) and the complexes examined in the electron microscope after negative stain (Fig. 6). VP19C can condense DNA into a structure with toroidal morphology (panel A, marked by black arrows). Most of the DNA structures condensed with full length VP19C have a similar toroidal morphology; the sizes of the toroids varied with a mean outer diameter of 9.1 nm and a standard deviation of 2.2 (n = 74), (Fig. 7). The structures of the NP complexes formed with VP19C N-ter^72^ (Fig. 6B) were different. The data show VP19C N-ter^72^ can induce the DNA molecules into a condensed state (panel B, black arrows) along with loop formations (Fig. 6B, white arrowheads). This morphology of the condensed DNA is different from the structures formed by whole protein and this could correlate to the different sizes of the two proteins. Measurements of a few of the loops induced by VP19C N-ter^72^ indicate the loop lengths of approximately 300 to 1000 base pairs. As expected no condensed morphologies were found in samples that had only DNA (panel D) or DNA with GST protein (panel C).

## Discussion

The present study demonstrates that purified VP19C can bind to and condense DNA molecules. However, this binding was not sequence specific in that VP19C can bind DNA of both viral and bacterial plasmid in origin. VP19C specifically binds dsDNA but does not bind ssDNA as observed in the gel shift assay. It was also observed that unlabeled dsDNA molecules can compete effectively with the labeled DNA molecules to bind with VP19C. Furthermore, nucleo-protein complex formation was inhibited by high sodium chloride concentrations, an observation that supports the notion that this interaction is non-specific [30] and that electrostatic forces play a crucial role in this interaction between DNA and protein. The congruence of all these observations indicates that the DNA binding property of VP19C is a non-sequence specific interaction. The DNA binding domain is located in the N-terminal domain (VP19C N-ter^72^) of the VP19C. This was confirmed by alanine-scanning mutagenesis which identified the importance of positively charged arginine residues in this N-terminal polypeptide for the DNA-binding activity.

When the nucleo-protein complexes were analyzed by ultrastructural methods a remarkable observation was made, that is, VP19C can condense DNA into a toroidal structure. The thickness of the VP19C induced toroids, which is inversely related to the extent of DNA condensation, is smaller than the thickness of toroids induced by a chemical agent like hexamine cobalt III [31]. The toroid diameter of DNA (3 kb) in the presence of hexamine cobalt III is on average 40 nm [31], whereas VP19C induced toroids (5.9 kb BamH1 K) have on average an outer diameter of approximately 9 nm. This indicates that the VP19C-DNA toroid is highly condensed and several molecules of DNA potentially wrap around the toroid such that the toroid grows both outward and inward, keeping the toroid diameter relatively constant. This is analogous to the viral DNA packaging inside the capsid [2], [32]–[34].

The formation of the ‘DNA-loop’ with VP19C N-ter^72^ is not surprising. In fact the first step in toroid formation is the spontaneous formation of a ‘DNA loop’ along a DNA substrate. This loop acts as the nucleation site for condensation on which the remainder of the DNA polymer condenses and leads to the formation of a proto-toroid [31]. It is possible that VP19C interaction with DNA reduces the DNA–DNA association energy such that proto-toroids are formed by the condensation of a single DNA polymer. The nucleation of DNA is happened in the first loops of the proto-toroid slide past one another to reduce the structural strain (from loop formation) in the condensed DNA and thereby increase their diameters, to eventually produce the full-grown toroids. Similar types of DNA loops have been observed during interaction of activation domain of the bovine papillomavirus E2 protein with DNA [35]. It is also possible that there are additional DNA binding sites in VP19C, however, sequence analysis indicates that the majority of the positive charges contributed by arginine residues are concentrated at the N-terminus of the protein (Fig. 1 and Table 1). It has been demonstrated that salmon protamine (arginine-rich basic protein) induced coiling of sperm chromatin or synthetic DNA into toroidal structures [36]–[38]. Similarly arginine-rich small peptides condensed the DNA to its toroidal structure more efficiently than protamine [38]. Therefore, condensation of DNA by VP19C appears to resemble protamine based DNA coiling.

The extent of condensation also depends on the charge density of the multivalent cationic ligands. VP19C is positively charged (pI = 9.06); however, most of the positive charges (mostly from arginine residues) are present at the first 72 amino acid residues (pI = 11.70), therefore, the charge density at the N-terminal domain is very high and we assume that DNA condenses at higher order. Not only that, the size of N-terminal domain of the VP19C is very small and therefore the resulting complex migrate longer distance compared to full-length protein (Fig. 5A). Besides, the complexes are very stable even at high dilution and are not disrupted in the presence salt (up to 200 mM NaCl, Fig. 4)). This DNA-binding property of VP19C may thus shed light on a potentially new physiological role for the protein in its natural environment.

This novel DNA binding property associated with VP19C N-terminus could be related to DNA encapsidation and stabilization. Of note, VP19C is found on the capsid shell only. The virus genome is tightly condensed in the capsid core and therefore there is high degree of repulsion between the different strands of DNA that come into contact with each other. VP19C could act in a manner similar to spermine by neutralizing the charge of the DNA allowing strands to pack more tightly with each other. Spermine has been shown to be associated with HSV-1 nucleocapsid, however, it was determined that the amounts present could only neutralize 40% of the virus genome [39]. Interestingly, inhibitors of polyamine biosynthesis had a greater effect on cytomegalovirus (a betaherpesvirus) replication than on HSV replication, which could correlate with the observation that this N-terminal domain is alphaherpesvirus specific [40]. If DNA stabilization is one of the functions of the N-terminus of VP19C then it would be expected that mutants in this domain would result in reduced levels of DNA-filled capsids or defects in DNA stabilization. There is evidence to support this in the phenotype observed in the mRFP tagged VP19C expressing virus. A large tag (mRFP1) at the N-terminus of VP19C in this virus was sufficiently exposed on the capsid surface for polyclonal antibody reactivity, while a small flu hemagglutinin (HA) epitope was inaccessible to the antibody [25]. This observation implies that the topology of the N-terminus of VP19C is directed towards the inner core of the virus, that is, the DNA core. Although the mRFP tagged virus plaqued on Vero cells, its burst size was significantly reduced by at least 15 fold. The growth defect appears to be at the level of encapsidation of capsids, in that, the levels of DNA containing C capsids were substantially decreased relative to non-DNA containing capsids [25].

Both the NLS and DNA binding domain reside in the N-terminus of VP19C. In both cases the positively charged arginine residues likely contribute to their respective functions. UL47 of HSV-1 has a similar arginine-rich NLS, which overlaps an RNA binding domain. The functional role of this domain has not been fully established but the data show UL47 binds with RNA and it acts as a nucleo-cyctoplasmic shuttling protein [41]. Random transposition insertions in the first 100 amino acids did not affect the interaction of VP19C with its binding partner, VP23. Spencer *et al*. [14] demonstrated in the recombinant baculovirus system that deletion of the N-terminal 105 amino acids did not affect triplex formation but did impair capsid assembly. Similarly Adamson *et al*. [7] showed that a domain between amino acids 45 and 63 was required for capsid assembly and virus replication. Therefore, in agreement with Adamson *et al*. (1), but in contrast with the prior understanding that the N-terminus is not required for HSV-1 assembly and replication, our data show that the N-terminus has an important functional domain that has yet to be identified.
